# The use of experiential knowledge in the role of a psychiatrist

**DOI:** 10.3389/fpsyt.2023.1163804

**Published:** 2023-06-15

**Authors:** Marjolein Boomsma-van Holten, Alie Weerman, Simona Karbouniaris, Jim Van Os

**Affiliations:** ^1^Division Neuroscience, University Medical Center Utrecht, Utrecht, Netherlands; ^2^Research Group Mental Health & Society, Windesheim University of Applied Sciences, Zwolle, Netherlands; ^3^Research Centre for Social Innovation, HU University of Applied Sciences Utrecht, Utrecht, Netherlands; ^4^Department of Public Health and Primary Care, Leiden University Medical Center (LUMC), Leiden, Netherlands

**Keywords:** psychiatrists, lived experience (of the illness), experiential knowledge, lived experience practitioners, disclosure

## Abstract

**Introduction:**

There is increasing interest in the use of experiential knowledge and the development of experiential expertise in mental health. Yet, little is known about how best to use this expertise in the role of a psychiatrist.

**Objective:**

This study aims to gain insight into the concerns of psychiatrists using their lived experiences with mental health distress as a source of knowledge for patients, colleagues and themselves.

**Materials and method:**

Eighteen psychiatrists with lived experience as patients in mental health care were interviewed with a semi-structured questionnaire. The interviews were analyzed using qualitative narrative thematic analysis.

**Findings:**

The majority of the respondents use their lived experience implicitly in the contact with patients, which makes the contact more equal and strengthens the treatment relationship. When explicitly using experiential knowledge in the contact with patients, thought should be given at forehand to its purpose, timing and dosage. Recommendations are that the psychiatrist should be able to reflect on his/her lived experience from a sufficient distance and should take patient factors into account. When working in a team, it is advisable to discuss the use of experiential knowledge in advance with the team. An open organizational culture facilitates the use of experiential knowledge and safety and stability in the team are vital. Current professional codes do not always offer the space to be open. Organizational interests play a role, in the degree of self-disclosure as it can lead to conflict situations and job loss. Respondents unanimously indicated that the use of experiential knowledge in the role of a psychiatrist is a personal decision. Self-reflection and peer supervision with colleagues can be helpful to reflect on different considerations with regard to the use of experiential knowledge.

**Conclusion:**

Having personal lived experiences with a mental disorder affects the way psychiatrists think about and performs the profession. The perception of psychopathology becomes more nuanced and there seems to be an increased understanding of the suffering. Even though harnessing experiential knowledge makes the doctor-patient relationship more horizontal it remains unequal because of the difference in roles. However, if adequately used, experiential knowledge can enhance the treatment relationship.

## Introduction

Approximately 40% of the Dutch population will experience a mental disorder during their lifetime (1). It is plausible and there are indications that this percentage is higher among healthcare professionals (2).

Novel policy attempts to reform the mental health field show several interesting developments and innovations in which the value of lived experiences of mental health professionals are discovered. Whereas the introduction of peer support workers in mental health services contributed to the transformation in the direction of a recovery-oriented landscape in general, it also consequently led to a division between professionals. Traditional professionals felt unseen and disadvantaged.

Nurses and social workers especially are speaking out about their own experiences with mental health distress, and some became inspired by working alongside peer support workers or lived experience practitioners, which facilitated coming outs about hidden personal histories with mental health distress (3).

This led to the further acknowledgement of the worth of lived experience in mental health settings. Yet, many other professionals felt urged to keep their personal experiences at bay. To date, there is very little scientific evidence on the professional use of this type of knowledge in mental health care. The Division of Clinical Psychology and the Royal Australian and New Zealand College of Psychiatrists value the lived experiences of, respectively, psychologists and psychiatrists, stating “lived experiences can provide a vital contribution to stigma reduction” [British Psychological Society, 2020 (4);]. Yet, psychiatrists are commonly not educated in recovery and resilience principles, nor in working with lived experiences professionally (5).

In the last 25 years, the value of knowledge gained through experience has been (re)discovered (6). The term experiential knowledge is coined by Borkman in 1976 which she defined experiential knowledge as the truth learned from personal experience with a phenomenon rather than truth acquired by discursive reasoning, observation, or reflection on information provided by others (7).

.Experiential knowledge may be a result of dealing with mental distress and disruptive life events and going through a recovery process by learning to reflect on it with others.

Being professionally open about personal experiences with a mental disorder is part of the professional use of experiential knowledge. Experiential knowledge contains, apart from one’s individual experiences, also collective knowledge, by discovering common elements through which lived experiences transform into experiential knowledge (8). Experiential knowledge especially concerns knowledge about ‘personal’ and ‘social recovery’ whereby you; rediscover yourself, connect, find meaning again, rediscover social roles as well as learn to deal with still present limitations, vulnerability or illness (9).

Since 2013, the profession of ‘experts by experience’ has been recognized and they are harnessing personal lived experiences while not being traditionally educated as a healthcare professional.

They did inspire the established healthcare workforce to become more open about their lived experience with disruption and recovery, as the latter were not mandated to use such expertise openly in their roles traditionally. With the introduction of the research project ‘The contribution of experiential expertise of professionals in care and welfare practices’ (9) and its follow-up, since 2017 healthcare professionals have already started to use experiential knowledge and professionalize it to experiential knowledge in their role as healthcare professionals. Experts by experience are often creative, they come up with different solutions and can encourage from their own experience and reduce the stigma which can promote participation (10). Among psychiatrists, there are different views on professionalism, which leads to different views on the use of experiential knowledge. Because of this, the use of experiential knowledge in the role of a psychiatrist seems to be scarcely accepted in the profession. To date, most studies have examined the use of experiential knowledge by social workers and nurses, while very few have looked into its use by psychiatrists (5, 11, 12).

More and more healthcare professionals in the Netherlands are being trained as lived-experience practitioners (13). Preliminary results from research demonstrated that therapists, including psychiatrists, lag behind (9). There is as yet no academic training aimed at developing experiential expertise, although a master’s degree is currently under development in the Netherlands. Psychiatrists seem to perceive suffering from a mental disorder primarily as vulnerability while recovering from a mental disorder requires a great deal of resilience and can therefore also be seen as a strength. The personal experience of and learning to deal with a mental disorder can be used as a valuable additional source of knowledge (14). Many care organizations have to get used to the idea that psychiatrists can also make use of this source of knowledge and that they too can use experiential knowledge.

### Objective

The purpose of the study is to gain insight into the concerns and opportunities when using experiential knowledge in the role of a psychiatrist. The ultimate goal is to improve the mental health care provided. Experiential knowledge is here defined as knowledge that the psychiatrist personally has gained, in the role of a patient with a mental disorder.

Sub-questions drawn up are:

1. What is the influence of experiential knowledge on professional knowledge?
2. What are the reasons to use or not to use experiential knowledge in contact with patients?
3. What are the reasons to use or not to use experiential knowledge in contact with colleagues?
4. What are the advantages and disadvantages of using experiential knowledge in (social) media?
5. What are the recommendations when using experiential knowledge for the psychiatrist?

## Materials and methods

**Figure 1:**
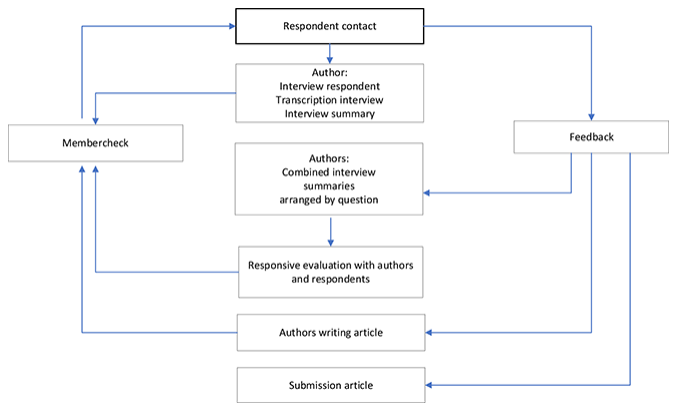
This research process was cyclic, data were collected, processed and collected again in a cocreation between authors and respondents.

Qualitative research was conducted using in-depth interviews and an interactive focus group. The data were processed with narrative thematic analysis, to do as much justice as possible to the respondents’ stories within the context and the meaningfulness of the whole. Respondents were given opportunities to refine and add to their input. They had an active role in processing the data (15). The aim here was to get to the heart of the matter in dialog and to answer the research question in such a way that it would be an accurate reflection of what the respondents meant. The data analysis was structured so that it could also be described systematically. It involved a cyclical research process, where data were collected, processed and collected again (Figure 1).

### Research population

The aim was to interview 20 psychiatrists because, with that number in this homogeneous group of respondents, a saturation of information was expected.

### Recruitment of respondents

At the spring congress of the Dutch Association of Psychiatry (NVVP) in April 2019, we presented a poster (16) and a discussion group “Being open about lived experience, I’m not crazy, am I?,” in which over 50 attendees participated. Afterwards, the attending psychiatrists were invited, to participate in this study.

### Ethical aspects

We realize that this is sensitive information and have taken care to anonymize the data. The study was approved in advance by the Medical Ethical Committee and respondents signed for informed consent.

### Data collection

The first part of the study consisted of a personal semi-structured (in-person) interview lasting approximately 60 min. The questions were formulated per sub-question (Appendix 1). There was room to ask further questions. Background information of the respondents was collected on LinkedIn in advance. As an introduction to the interview, the work experience of the respondent was discussed. To ensure data reproduction, the interviews were recorded and afterwards transcribed. These transcripts were anonymized and used as raw data for the follow-up steps in the data analysis process. The researcher’s insights from the interviews were recorded in a separate file, as this would be potentially useful information for the discussion.

### Data analysis

The data analysis process proceeded through several stages in line with narrative thematic analysis methods (17, 18). Figure 1 shows how the data were collected structurally and analyzed in stages.

Each interview was first summarized to improve the text in readability and size and thereby frame the data. Storing interview citations as narratives with the summary preserved meaningful context. The summaries of the interviews were then organized by research questions. In this way, similarities and differences between respondents as well as salient information became readily apparent. The next step was to make a summary per research question while keeping the source traceable. Based on this, the results were presented in a PowerPoint presentation.

Respondents were invited for a responsive evaluation with the researchers, where the PowerPoint presentation was discussed step by step. In a group of four respondents, outcomes were supplemented, refined or provided with critical comments. The outcomes were enriched in this half-day group meeting. At the end of this responsive evaluation, the outcomes and the relations found were filed in a report, with illustrative narratives chosen to preserve meaning and context. Efforts were made to ensure that the text was readable even without the narratives.

### Credibility and confirmability

First of all, all interviews were conducted semi-structurally, with the questions prepared in advance in joint consultation with the co-authors. Questions were partially inspired by the knowledge gap identified at the conference and in the literature review.

The interview provided sufficient space to address issues that could not be anticipated in advance so that valuable relevant information was not left out. This contributed to the credibility of this research.

In addition, three member checks took place as part of the data analysis procedure.

1. All respondents were asked to check whether the summary of the interview accurately reflected what they intended to say and to adjust it if necessary. All fifteen respondents indicated that the summary was a good reflection of the interview.
2. A 3-h responsive evaluation followed together with 4 respondents to look for connections, shared reflections, and meaning and to refine the outcomes.
3. Finally, all respondents were asked to read and comment on the article before the manuscript was submitted. This did as much justice as possible to what the respondents meant to say.

Subsequently, several peer checks took place during this study. The method of the study, as well as the interview questions, were jointly drafted. A peer check took place when summarizing the interviews, where the first two interviews were independently summarized by two researchers to determine the degree of overlap. These interviews were very rich in information and very different in content. The summaries differed little from each other and thus reliability was adequate. The remaining 16 interviews were then summarized by one researcher.

Three authors were involved in combining the interview summaries with a question and looking for similarities and striking information among the respondents. All four authors participated in the responsive evaluation and jointly wrote the article, selecting the illustrative narratives.

### Respondent selection

A total of 20 psychiatrists were interviewed, of these, 17 psychiatrists were recruited at the spring conference and three psychiatrists were approached from their network. Ultimately, 18 interviews were used for data analysis. During the interview, two respondents were excluded because they appeared to have no lived experience with a mental disorder.

**Figure 2:**
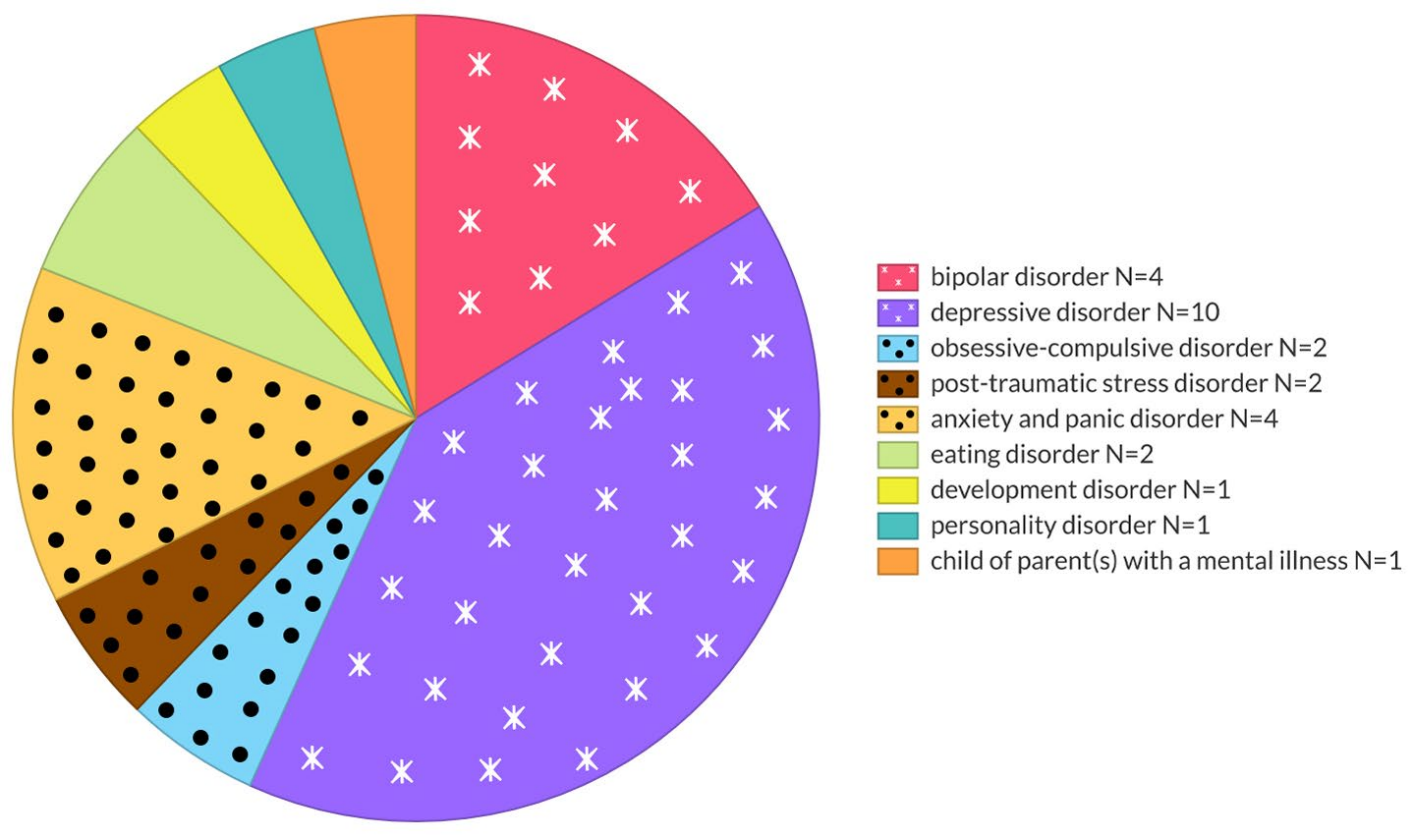
Personal experiences with mental disorders concerned various mental disorders.

Respondent’s personal experiences with mental disorders (Figure 2) were diverse and some respondents had dual diagnoses. It concerned the following disorders:

- bipolar disorder (BD) *N* = 4 and depressive disorder (DD) *N* = 10 (clustered as mood disorders).
- obsessive compulsion disorder (OCD) *N* = 2, post-traumatic stress disorder (PTSD) *N* = 2, anxiety and panic disorder (AD) *N* = 4 (clustered as anxiety related disorders).
- eating disorder (ED) *N* = 2, developmental disorder (DD) *N* = 1, personality disorder (PD) *N* = 1 and child of parent(s) with a mental illness (COPMI) *N* = 1.

In total, 11 adult, six child and adolescent and one elderly psychiatrist were involved. They work throughout the Netherlands. Two of the participating psychiatrists were treated by a psychiatrist who used experiential knowledge in the treatment of these respondents.

**Figure 3:**
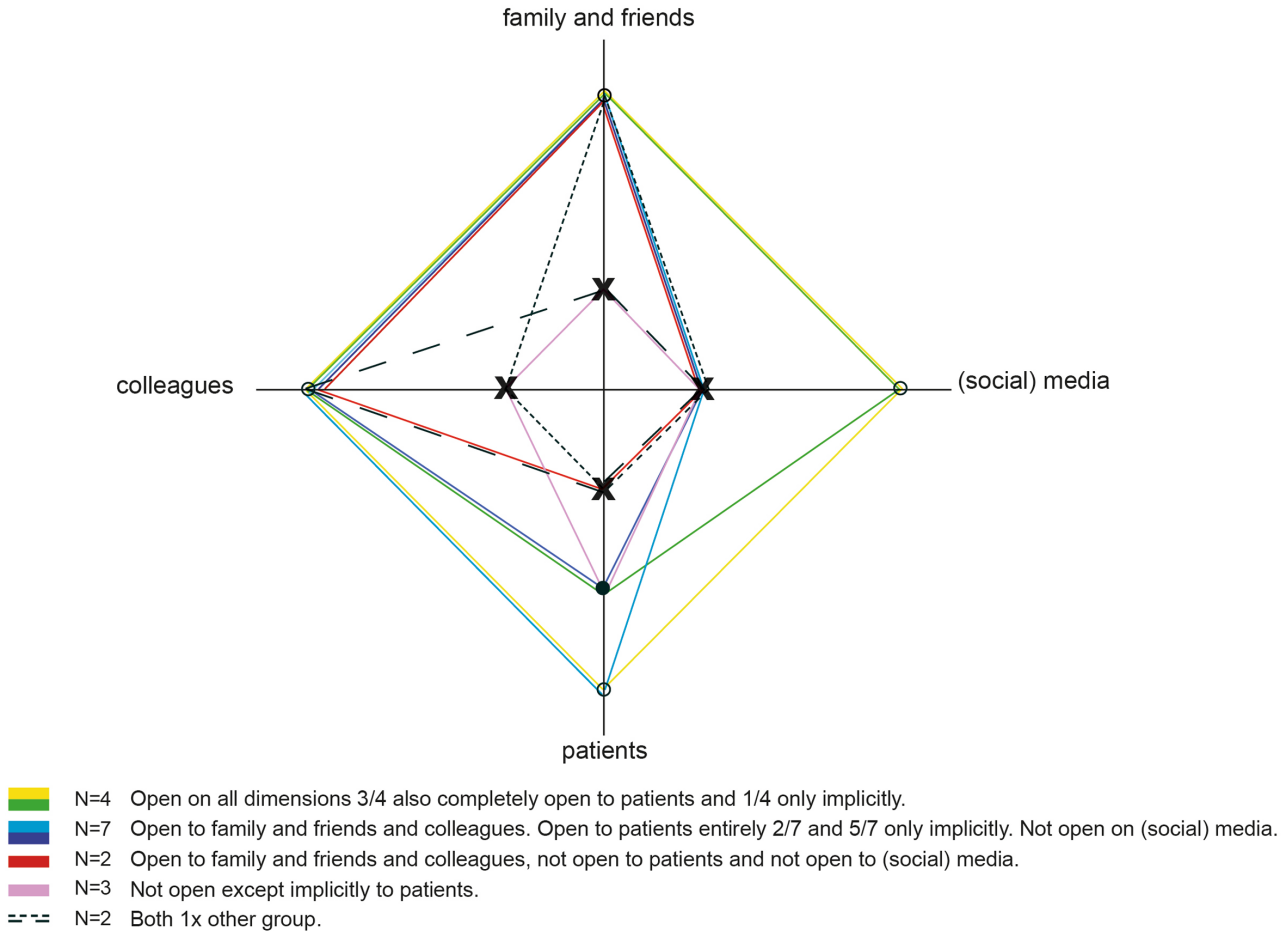
Domains on which respondents are open about personal lived experience with mental ill-health. X is not open about personal lived experiences • only implicit ° explicitly open.

Of all psychiatrists, four were open about their experiential knowledge in the (social) media, 14 were open to colleagues and five psychiatrists used their experiential knowledge explicitly with patients in their role as a psychiatrist, nine psychiatrists did so only implicitly. Two psychiatrists showed a very critical attitude toward the use of experiential knowledge in the role of a psychiatrist but wanted to participate anonymously in this study so that their knowledge and experience could be included (Figure 3).

## Results

### Influence of experiential knowledge on professional knowledge

Lived experience and its derived experiential knowledge affect the psychiatrist and his or her (perception of) professional knowledge.

First of all, respondents’ personal lived experience with mental distress changed their thinking about mental disorders. It is socially taboo to talk about having a mental disorder, while it can happen to anyone. As a result, psychiatrists experienced increasing problems when getting ill. From this perspective, respondents realized the relevance of breaking the taboo.

*Interview 104: You cannot talk to each other at all anymore about the miserable side, it all has to be great, fun and cool. So apparently an idea has arisen that everything is super-makeable, feeling unhappy is not part of it and that has to go.*

Secondly, respondents indicated that they have become more aware of the spectrum between illness and health, and determining when someone suffers from a mental disorder is more difficult because of this. From there, respondents also developed a more nuanced view of treatment. It is important to know guidelines and to work evidence-based, but you can deviate from this flexibly to your patient’s advantage. For example, from their own experiences, respondents appreciated it when the practitioner searched together to find the most individually appropriate treatment.

*Interview 111: I can think of what has helped me, but I know that you really have to tailor what fits someone and what they need.*

Involving family and friends has become more important to respondents. A mental disorder affects relationships, thus when family and friends are well supported also the patient benefits. Furthermore, respondents’ lived experience with a mental disorder has led to an increased appreciation for peer contact, e.g., one respondent highly valued peer contact when he received group treatment.

Consequently, in their clinical practice respondents have become more relationship- and person-oriented instead of disorder-oriented than usual in working according to the DSM. The patient’s story has become more central, where the most important question is not what symptoms there are, but what the suffering is caused by.

Thirdly, the majority of the respondents indicated that lived experience implicitly influences contact with patients, even if the psychiatrist does not tell them that he or she has been ill as well.

Respondents’ attitudes toward patients also changed because the contact has become more equal.

*Interview 108: I think experiential expertise can be used especially in your attitude toward your patient. So I do not have someone sitting across me who has something I’ll never have, because I’m too good for that, too healthy. No, I’m sitting opposite someone who is just like me, knee-deep in mud and has to get through the viscousness of existence, and that determines my attitude.*

From their patient experience, respondents indicated the importance of building trust before starting treatment. It can be a big step for a patient to ask for help.

*Interview 104: When you are out of control yourself and then you have to ask another person for a helping hand, really I thought that was super scary. The other person can destroy you. So then you have to have the courage to trust in the goodwill of someone else.*

Based on their lived experience, respondents were better able to put themselves in the position of the patient’s suffering, which made it easier to connect with the patient. Respondents indicate that offering a fix is not always the best solution. It is also important to create space so that feelings such as sadness and anger are acknowledged. This creates a more intuitive understanding of the problem, following a quick quality improvement of the contact.

*Interview 120: The patient: ‘yes I always felt it. You understood it all very easily.’ So yes, often I think there is a good click because they understand that you understand… and that gives a better doctor-patient relationship and that is also worth a lot. Then you do not have to say what it is exactly.*

Also, the respondents stressed they recognize internal conflicts and certain behavior patterns more quickly, which also creates a stronger connection. At the same time, respondents attach to the maintenance of patient autonomy and control, which made them pay more attention to this specifically in the psychiatrist-patient relationship. They see more recovery possibilities, as long as one finds a suitable place that supports development.

### Reasons to use or not to use experiential knowledge in contact with patients

Different considerations were mentioned about harnessing experiential knowledge with patients.

First of all, respondents indicated that the most important reason to explicitly use experiential knowledge is that they think it serves a purpose that benefits the patient, which is mainly strengthening the treatment relationship. By being explicitly open, common ground is shared, by which the patient recognizes something of his own situation in the story of the psychiatrist. This strengthens the working relationship and solidifies the contact.

*Interview 104: Recognition is recognition. And recognition already does so much. If you talk to each other about something you have both been through, sometimes that already creates such a powerful bond, but that also gives recognition, the feeling of recognition, of the other person seeing me. And that all sounds very easy, but anyone who has experienced it themselves will tell you how extremely valuable that is. You cannot put that into words at all.*

In some cases, the psychiatrist’s disorder was recognized by patients before mentioning it explicitly. This motivates to be more open about lived experiences as one can sometimes not avoid them. Involuntarily and implicitly one has already revealed things.

Secondly, respondents indicate that the treatment relationship is established more quickly and patients experience more proximity in the contact. From this common basis, the situation can be assessed collaboratively and the expectation of what the psychiatrist can contribute becomes more realistic while the patient’s autonomy is enhanced.

*Interview 104: Because of course it’s about, it is literally standing next to someone. And maybe keeping that person out of the wind or rain, but after that, you have to walk yourself. And you can have that conversation much more easily with each other because you are not sitting across from each other.*

Thirdly, respondents also indicated that they wanted to be a role model in showing how you can live with your disorder and talk about it openly, without being rejected. It can even be a strength if you dare to be vulnerable. Also, experiential knowledge can be used explicitly to reduce shame. In doing so, it has a destigmatizing impact.

*Interview 120: They are happy that I am also a human being and that I can also have something, they do not feel so idiotic, they feel less ashamed and less ill. It destigmatizes, it connects, they are happy and feel seen and heard and understood... you can say this too because you know how it is yourself.*

Consequently, patients may experience that they are not alone. It can then also provide hope when patients see one can function well, even with a certain condition.

*Interview 114: That the patient can get hope. While they themselves think ‘oh now everything is over for me, I can never become anything again. End of all my dreams and all my developments’ and so on. Then it can be useful to see that you can live with that because I’m quite normal.*

Few respondents stated that in some cases self-mockery is practiced, which allows them to laugh about themselves, while it resembles a similar situation of the patient. Accordingly, space is created to look at the patient’s situation differently.

*Interview 104: Because of this way of working, I can also joke a lot more with clients. That you actually just kind of ridicule your own vulnerability and how you deal with it. And that is so recognizable. It also puts things into perspective.*

Fourthly, some respondents indicated that they harness their lived experiences, to confront the patient with a matching situation of their own. Respondents who frequently use experiential knowledge in contact with patients indicate that such disclosure is very much appreciated by patients, which worked stimulating to use experiential knowledge more explicitly.

*Interview 104: Because it is almost always greatly appreciated that you dare to show a part of yourself. So patients are very accommodating and really see your good intentions. A lot of people really appreciate it.*

Lastly, several respondents also used experiential knowledge explicitly to influence patients’ readiness and motivation for treatment.

*Interview 105: If I think it benefits the contact. For example, if patients are hesitant about medication. Then I say ‘I understand that very well, but I started using them myself’. Or that theoretically I do understand how you should do an exposure, but how incredibly difficult and difficult it is to expose yourself, that terrible anxiety you feel.*

Conversely, there were also reasons mentioned for not harnessing experiential knowledge.

First of all one respondent indicated that a psychiatrist is not mandated to be open about personal lived experiences, since this is not his or her task.

*Interview 103: My strength Is In diagnostics and directing.*

Secondly, several respondents believe that if one is still in training, openness about one’s mental condition is not advisable. You are in a dependent position as a resident and on top of that you still have to accommodate the profession.

*Interview 119: I could not have done this ten years ago, because then you are so much more dependent on others.*

Thirdly, shame or fear was also mentioned as an important reason to not be open. There is fear of not having enough control over one’s feelings and boundaries.

*Interview 104: Yes if I start talking about something that affects me so much that it overwhelms me... I do not do myself any favors and neither does the other person.*

Additionally, there is also fear of disapproval, fear of distortion of information and in the worst case a disciplinary measure.

*Interview 102: The misery is that once I have said it, I cannot turn back. I’ve never had a disciplinary case. But a complaint does come up once in a while (..) I realized, I’m sharing something and it ends up with someone in a different state, or in an extreme sense, who has bad intentions or wants something different with it. In this digital age, I cannot undo what I say. I find that a difficult thing. So that makes me a bit more reserved.*

Fourthly, being open about experiential knowledge should not stem from the satisfaction of one’s own needs, as seen in narcissism or exhibitionism. Nor should openness come from a disorder, as associated with hypomania or complicated grief. The intention to use experiential knowledge has to be right, otherwise, you should not do it.

*Interview 113: That you say things out of self-interest. The other person should get something out of it. It should not be something because you want to show what wise words you can speak.*

Fifthly, some respondents mentioned that there are treatment settings or therapy modalities that hinder the use of experiential knowledge. Also, the culture of the organization can be unsuitable for its explicit use, even though in recent years more openness has been established. Forced settings, such as a crisis department or forensic mental health care, are found to be less suitable for using experiential knowledge.

*Interview 116: In difficult therapies. It’s about his or her story, his or her theme, his or her words, his or her feelings and not so much my stuff. It’s seen in analytic therapy, a little bit as acting-out of the therapists.*

Lastly, several patient-bound reasons are cited that make psychiatrists more reluctant to explicitly harness experiential knowledge. For instance, when patients find it difficult to empathize with the other person or, on the contrary, do so too much. They are also more reserved with patients who have difficulty with boundaries or when there is a risk of the relationship merging.

*Interview 104: And because of that, the attachment and bonding become much more powerful, but it can also become too powerful so you have to start watching those boundaries again. And that the other person does not become involved with you. Involved is a very beautiful word for fusion. And that’s what I mean, again, that intuitive sense, if you notice that patients have trouble with that, then that can really be a reason not to do it.*

When a patient, motivated by resistance, asks about the psychiatrist’s experiences, patients are also more reticent to answer this question directly; it is better to find out first where the resistance is coming from.

Also, psychiatrists are more cautious about explicitly using experiential knowledge in contact with a child or an elderly person. In this regard, the degree to which the patient can identify with the practitioner seems to play a role.

*Interview 115: Generational differences... That also has a bit to do with the expectation patterns from the patient, and how they see the doctor.*

### Reasons to use or not to use experiential knowledge in contact with colleagues

Different considerations were mentioned about harnessing experiential knowledge with colleagues. First of all, some are related to the culture of the organization. An organizational culture which is characterized by openness and sufficient safety and stability in the team, makes it easier to be open about lived experience, while rapid staff changes can bring about the opposite.

One of the respondents asked a colleague to keep an eye on her so that early symptoms are recognized earlier. Hence colleagues can help, assist or think along in difficult situations when being explicitly open about experiential knowledge.

*Interview 119: But with crisis assessment around depression and so on, I have to be very careful and so sometimes I do not do that. Because that’s so unpleasant, I did have a few times when that just got too intertwined and I thought, ‘I cannot do this now.’ You need distance for your crisis assessment, you need a certain kind of distance to be able to assess properly. And I could not do that. And because I had already been open to my colleagues they took over.*

Another respondent was approached by a colleague who recognized that she had a mental disorder. When the colleague mentioned this, she noticed that it was better to speak openly about it, because you work together as a team and you have to be able to rely on each other.

*Interview 115: You can deny that something is going on, but people still see it.*

Secondly, team factors are mentioned to explicitly deploy experiential knowledge in the team. The explicit use of experiential knowledge can initiate a team discussion, in which colleagues become aware of the way they think about patients and the influence of patients on them. Bringing in experiential knowledge can promote (self-)reflection and awareness.

*Interview 119: I said, Yeah you know, I just notice, I just identify with them too much. I’m shocked at the prejudices that I had myself. I just, I notice now that I protect them too. What about your prejudices? And that was a really nice conversation, just with the team.*

Experience knowledge can also be harnessed to destigmatize and stand up for injustice within the team. It can also be a goal to include experiential knowledge in the team to make it easier for colleagues to discuss their vulnerabilities with each other and to promote experiential expertise.

*Interview 119: Since I’m open, there are a lot of staff and colleagues who are also open to me. And with whom I’ve worked for years and from whom I never knew that because it just wasn’t about that. And it’s just mutually very nice that you can now be open about it. What I find very nice, especially in the department where I now work, is that it’s really becoming more normal. Not only toward me but also among all colleagues. That you are open about your own mental health.*

Conversely, there were also reasons mentioned for not harnessing experiential knowledge.

Firstly, the most important reason for staying reserved about disclosing personal lived experiences has to do with one’s specific mental health condition. Not being fully employable for irregular shifts is frequently mentioned, as this can be a stressor in psychoses and mood disorders, or is hard to combine with antipsychotics.

In times of severe budget cuts and financial scarcity, organizations pay particular attention to the production and quality of care provided by psychiatrists. Openly using experiential knowledge and thus being open about having a mental disorder, brought six respondents into a conflict situation with their employer, resulting in job loss. Hence, respondents stress a sense of security in teams is paramount.

*Interview 118: It has to feel safe for me to talk about this. Because this remains, and now too, emotionally taxing. That does not work either when you do not feel a certain connection. And if I do not feel that connection, then it does not feel safe for me to be able to share this.*

Secondly, There has to be a clear motivation to use the knowledge gained through experience explicitly in the team. It’s important to think carefully about the timing, the purpose, the way and the dosage of experiential knowledge.

*Interview 102: I’m not going to just start telling my story. I would not know why anyone should hear it necessarily.*

Respondents indicated one should not go beyond the limits of colleagues when explicitly harnessing lived experiences.

*Interview 108: I still think that colleagues have a certain tolerance limit toward each other, that at work the contacts are mainly functional. They are not friendships, they are functional contacts and as soon as you leave you are forgotten... You have a common interest, which is that you cannot manage on your own. You need each other and so you have to have a good relationship.”*

Thirdly, within the physician culture, hard work is valued and high standards must be met at a young age, even before starting medical school, to enter training. This curves a certain type of physician who is highly demanding and also affects the professional identity and professionalism. There is strong condemnation toward each other if you cannot work according to the norm, e.g., due to a mental disorder.

*Interview 118: For a long time I looked at doctors as if they are kind of demigods and not vulnerable and they are untouchable and they can do anything.*

A psychiatrist is expected to be psychologically strong and good at managing emotions and conflicts, within themselves, in contact with patients, the team and at the organizational level.

*Interview 107: As a psychiatrist, you have to be a model of emotion and conflict management.*

Lastly, since psychiatrists often carry end responsibilities, colleagues come to them when there is uncertainty. It is then more difficult to show your lived experience and vulnerability since it may undermine your position in the team and may lower interpersonal confidence.

### Advantages and disadvantages of using experiential knowledge in (social) media

Different advantages and disadvantages were mentioned by the four respondents who were open about their experiential knowledge on (social) media.

First of all, one of the mentioned advantages of exposing experiential knowledge on social media is to be able to act as a role model in creating openness and contributing to destigmatization.

Also, it’s easier to come into contact with colleagues who have similar lived experiences.

*Interview 102: It does open up something. It offers a perspective for colleagues to start talking about it. Which I absolutely did not expect as a result.*

Secondly, those who were open on social media indicated that contact with patients became easier after that. Patients can read as much as they want and therefore choose the time and the amount of information. In this way, the psychiatrist does not have to tell his or her personal story and may focus on how it is received by patients, or let them come up with more in-depth questions based on what they have read.

*Interview 105: But because of that, of course, my patients know in advance that I have it too, and that already makes them approach me differently. I get a whole lot of confidence with them, in advance.*

Conversely, respondents also mentioned the disadvantages of disclosing personal lived experience on social media, such as the increase in interpersonal contact and therefore having difficulties in keeping boundaries.

*Interview 105: It gives me something of belonging and connectedness as a peer. Sometimes I’m glad it’s at the end of my career. Because in some ways the contact becomes more intense, but your involvement, to keep it within sustainable limits I think also becomes more complex. Distance also has an advantage, of course, that you do not take everything home with you. So in that sense, I think it has become more intense, which I certainly like, but I also think ‘we must realize that proximity can also have disadvantages’.*

### Recommendations when using experiential knowledge

Recommendations on harnessing experiential knowledge with patients and colleagues were given.

To harness experiential knowledge with patients, the following recommendations were given:

1. It’s vital that a psychiatrist can look at his or her problems from a distance and view the situation on a meta-level before harnessing experiential knowledge.
2. Build up a relationship with the patient and only harness experiential knowledge when useful.
3. Think about the goal, timing and dosage of sharing in advance.
4. Even minimal disclosures about personal experiences may have a large positive effect on the relationship.
5. When a patient is treated in a team setting, it’s important to discuss the use of experiential knowledge with colleagues.
6. Be realistic and non-judgmental when serving as a role model, as every individual is unique.
7. Carefully assess in advance whether sharing experiential knowledge is appreciated. Ask permission before sharing and check afterwards how the information is received.

Harnessing experiential knowledge in the role of a psychiatrist is a personal choice and not part of the job. Therefore, providing good standard care is always the priority.

With regard to harnessing experiential knowledge with colleagues, there is a risk of tension in the team that might arise as colleagues consider the lived experience as something negative. Moreover, there is also often already a vulnerability inherent in the mental condition. Several respondents stress the importance of ways to protect oneself and personal vulnerability by:

1. By not telling everything and making choices in what you will and will not share.
2. By not sharing current issues that you are struggling with at the time.
3. When triggered, first apply self-reflection and think about whether you can and want to use knowledge from your experience.
4. By preparing yourself well and discussing the use of experiential knowledge- for instance in a peer supervision group- before using it.
5. By using experiential knowledge only in individual contact so that group dynamics do not play a role.
6. By asking colleagues to be careful with the information, because you do not want everyone to have this information.
7. Being cautious in using (social) media as they may be passed on incorrectly.

## Discussion

Speaking out about personal lived experiences often starts after being inspired by colleagues who are open about personal struggles. Respondents in this study developed their personal lived experience into experiential knowledge by sharing and trying out to harness insights with patients and colleagues and thereafter reflecting on it. Having gone through a mental disorder influences the way the psychiatrist thinks about and performs his profession. Having lived experiences as a patient influences the development of the personal-professional identity. While generally there is little room to fit a patient role into the professional, it is seen as courageous to do so (9, 12).

Many psychiatrists look at their own and each other’s mental disorders from the medical model, which is a disorder- and deficit oriented. As a result, having a mental disorder is basically seen by them as vulnerability (19). From the existing perception on professional identity, it is difficult to tolerate illness (5, 9).

Even though lived experiences are often part of the decision to pursue psychiatry, psychiatrists often feel insecure to disclose and worry about ramifications from colleagues and educators. Contractionary to social workers who were stimulated to reflect and discuss those reflections with patients as part of a deliberative practice, psychiatrists seem to be caught in reservations stemming from the psychoanalytic tradition.

Following additional training to professionalize experiential knowledge, will possibly help to look at themselves, each other and patients in a different way, which has a destigmatizing effect.

However, investments in primary curricula and medical culture are paramount. Little attention in the current curricula is paid to the emotional landscape of medical students, leaving those struggling with personal issues, isolated (20). Few psychiatrists choose to professionalize and use their experiential knowledge at the moment, as this is not yet part of the standard of care.

The personal choice to put experiences to use should remain compatible with the professional relationship between a psychiatrist and a patient. The findings of this study, show that some respondents began to see the psychiatrist-patient relationship as more equal, resulting in improved contact. However, the professional relationship remains unequal because the psychiatrist and the patient continue to have different roles in the contact, which means that there remains a difference in position, dependence and power. It is possible that in the future the use of experiential knowledge may also become part of standard care, as is already the norm in some psychotherapy traditions, e.g., existential psychotherapy.

Interestingly, the Diagnostic and Statistical Manual of Mental Disorders (DSM) seems to lose value among respondents, which may conflict with the professional role execution concerning the choice of treatment and financing of care. Even though working from frameworks such as the DSM is losing its value, it does not exclude experiential knowledge. The dominance of the medical model is currently decreasing in the Netherlands, because there is too little attention to personal meaning-making, for the life history and the social environment of patients. However, evidence-based methods are increasingly promoted and are part of the Dutch finance system.

The aim of this study was not to evaluate the patients’ satisfaction or the effect of using experiential knowledge. Appreciation of patients upon the use of experiential knowledge by healthcare professionals has been demonstrated in earlier research (21).

This research initiated the set up of a specific peer supervision group for psychiatrists. It is advisable to further professionalize lived experiences in peer supervision settings so that dilemmas that may occur when harnessing experiential knowledge can be discussed with colleagues.

To disclose to patients, one should be open to colleagues first, which is not without risks, as long as the professional code of conduct and culture has not changed (12). Analytical psychotherapy warns against ‘acting out behavior’ in psychiatrists to prevent the transgressive behavior of the therapist.

A striking observation during this study was the effect of the respondents’ stories on the researcher herself. Hope and confidence arose in the first author, who also has lived experience with a mental disorder. Despite a certain background one can still do the work as a psychiatrist well. By creating space for experiential knowledge, the personal and the professional identity can further integrate and/or balance, whereby the existing taboo on being open about personal vulnerabilities can be broken (16).

This study shows that this professionalization is necessary, and there are many considerations involved with the use of experiential knowledge It takes time, reflection and deliberation to learn to use lived experience in a professional way. Peer supervision with colleagues with lived experience helps to consciously reflect on the influence of personal experience in contact with patients and to professionalize experiential knowledge. Provided that it is used in the right context and with a legitimatized goal, it may contribute to contact with patients.

In the Netherlands, there are now four peer supervision groups for psychiatrists with lived experiences and it is being investigated how this could be further structured and professionalized in collaboration with the Netherlands Psychiatric Association (NVvP). It would be helpful if the Netherlands Psychiatric Association, like the Royal Australian New Zealand College of Psychiatrists, would support the development to further professionalize experiential knowledge among psychiatrists (4).

There is also a need for the development of a theoretical framework to apply experiential knowledge. Respondents mentioned that the use of experiential knowledge could possibly be in line with the framework and therapeutic modalities that use self-disclosure and lived experiences in a well-considered way, like in existential psychotherapy (22). When it becomes more common in the organizational culture or among colleagues to show more of the person as a psychiatrist, there will reasonably also be more space for using experiential knowledge. It is also indicated that following an open dialog training can help develop and use experiential knowledge (23). By learning to work together with each other as a team in an open dialog with patients strong emotions and uncertainty are tolerated in a crisis, as new meanings can be found over time, stimulating recovery.

This study contributes to the professionalization of the use of experiential knowledge by psychiatrists, as it identifies specific considerations and recommendations.

### Limitations

This study is innovative because no such research has been done on the use of experiential knowledge by psychiatrists. Yet, we recognize the following limitations of the current study: it was conducted in the Netherlands and there are no similar comparable international studies known. It was small in its size with a homogeneous group due to its qualitative nature and therefore may lack generalizability. In addition, respondents volunteered after attending the workshop or viewing the poster presentation, which may have led to a selection bias. They were interested beforehand in how lived experiences and their former patient identity could be used in the role of psychiatrist. By this selection of respondents, it is plausible that criticism with regard to experiential knowledge was less mentioned, although two of the respondents were against the use of experiential knowledge in the role of a psychiatrist, which has increased the reliability of the study.

The credibility of the research is high because, in all steps of the research process, close cooperation with the respondents took place, serving as a member check. In doing so, respondents indicated that they recognized themselves well in the data.

After 15 interviews saturation occurred, and during the data analysis of the last three interviews, no new information surfaced. This means that the number of interviews was sufficient to answer the research questions. Illustrative quotes from all interviews were used to do as much justice as possible to the diversity of stories of the respondents within the context.

## Conclusion

Having personally experienced a mental disorder affects the way the psychiatrist thinks about and performs his profession. There is more gradual thinking about mental disorders. On the one hand, there is more understanding of the suffering and the fact that life cannot be made, but on the other hand, they also see (more) opportunities for recovery based on experiential knowledge.

Almost all respondents use experiential knowledge implicitly in their contact with patients, even while they consider this to be a personal decision. In contact with patients, the doctor-patient relationship becomes more horizontal, even though it still remains unequal because of the difference in roles.

When the correct timing and dosage of the experiential knowledge are taken in to account and personal experiences are flexibly shared as is helpful for the patient, the treatment relationship can be strengthened and deepened. Recommendations are to look at personal lived experience with sufficient distance and to take patient factors into account. When working in a team, it is advisable to collectively discuss the use of experiential knowledge in advance.

Harnessing experiential knowledge in contact with colleagues, requires an open organizational culture, and sufficient safety and stability in the team. Considering current professional codes there seems to be more room for the use of lived experience, but the space is still limited as its associated with the burden of being ill.

The great advantage of exposing lived experience in (social) media is destigmatization and breaking the existing taboo’s. When being open on media, it’s important to monitor personal boundaries in the extent of information provided Especially in individual contacts with patients, this may play a role. Self-reflection and dialog with colleagues may help to guard boundaries and well-being.

## Data availability statement

The raw data supporting the conclusions of this article will be made available by the authors, without undue reservation.

## Ethics statement

The studies involving human participants were reviewed and approved by Mondriaan- committee scientific research. The patients/participants provided their written informed consent to participate in this study.

## Author contributions

MH, AW, and JO contributed to conception and design of the study. MH organized the database and datacollection and wrote the first draft of the manuscript, to which all other authors added. MH, AW, and SK contributed to the analysis. All authors contributed to manuscript revision, read, and approved the submitted version.

## Conflict of interest

The authors declare that the research was conducted in the absence of any commercial or financial relationships that could be construed as a potential conflict of interest.

## Publisher’s note

All claims expressed in this article are solely those of the authors and do not necessarily represent those of their affiliated organizations, or those of the publisher, the editors and the reviewers. Any product that may be evaluated in this article, or claim that may be made by its manufacturer, is not guaranteed or endorsed by the publisher.
