# Supplementary material for: The use of experiential knowledge in the role of a psychiatrist
Source: Front Psychiatry. 2023 Jun 15;14:1163804. doi: 10.3389/fpsyt.2023.1163804 (PMC10308280; doi:10.3389/fpsyt.2023.1163804)
Supplement: Supplementary file 1 [file Table_1.DOCX]

**Appendix 1**

Interview questions were prepared in advance for the semi-structured interview.

1. **Patient role data:**
a. What experiences do you have with a mental disorder yourself?
b. When was this, before, during or after your training as a psychiatrist?
c. What did the treatment look like?
d. What did having these experiences mean to you in terms of family, education, and work?
e. In what context are you open about your own experiences? What do you share and what not?

2. **Data dual role: what you do with patient experiences in the role of psychiatrist:**
a. To what extent have your own experiences influenced your thinking about professional knowledge and its use in practice as a psychiatrist?
b. Have you ever revealed anything about your lived experiences in contact with patients?
When did you do so? What was the effect of your self-disclosure on your contact with patients?
c. Also, have you ever intentionally not disclosed your own experiences? What were the reasons for not doing so?
d. Have you ever not revealed anything about your own experiences, while in retrospect you think you should have? What considerations played a role in this?
e. Have your experiences, influenced your way of dealing with colleagues?
f. Have you ever revealed anything of your own experiences in contact with colleagues?
g. What was the effect on your contact with colleagues? Was there any difference in how patients, applied professionals, psychologists, and psychiatrists reacted to your coming out?
h. Also, have you ever deliberately not disclosed your own experiences? What were the reasons for not doing so?
i. Have you ever not disclosed your own experiences, while in retrospect you think you should have? What considerations played a role in this?
j. In what ways did you develop in using your lived experiences?

3. **What considerations do you have in using your lived experiences for:**a. yourself and your own vulnerability?
b. for the client?
c. for colleagues?
